# Effects of Strength and Speed Training Programs on Physical Performance Variables in Futsal Players: A Systematic Review and Meta-Analysis

**DOI:** 10.3390/jfmk11020170

**Published:** 2026-04-24

**Authors:** Oscar Villanueva-Guerrero, Bruno Travassos, Hadi Nobari, Rafael Albalad-Aiguabella, Elena Mainer-Pardos

**Affiliations:** 1 Health Sciences Faculty, Universidad San Jorge, Autov. A23 Km 299, Villanueva de Gállego, 50830 Zaragoza, Spain; ovillanueva@usj.es (O.V.-G.); ralbalad@usj.es (R.A.-A.); 2 Research Center in Sports Science, Health Sciences and Human Development, CIDESD, University of Beira Interior, UBI, 6201-001 Covilhã, Portugal; bfrt@ubi.pt; 3 Portugal Football School, Portuguese Football Federation, 1495-433 Oeiras, Portugal; 4 LFE Research Group, Department of Health and Human Performance, Faculty of Physical Activity and Sport Science (INEF), Universidad Politécnica de Madrid, C/Martín Fierro 7, 28040 Madrid, Spain; hadi.nobari@upm.es

**Keywords:** training, jump, sprint, power, performance, team sport

## Abstract

**Objectives**: The primary objective of this systematic review and meta-analysis was to analyze the effects of training programs on different parameters of physical performance in futsal players. **Methods**: Following the Preferred Reporting Items for Systematic Reviews and Meta-Analyses (PRISMA) statement, a systematic search was conducted using PubMed, Web of Science, and SportDiscus databases. The search was conducted for the studies published between 2014 and 2024, and 13 studies were selected that met the inclusion criteria. The random-effects model with inverse variance weighting was used for the meta-analysis. Effect sizes (ES) were reported as standardized mean differences and presented with 95% confidence intervals (CI). **Results**: The effects of such programs showed a primary small effect size for vertical jump (ES = 0.38; 95% CI = 0.11, 0.64; Z = 2.76; *p* = 0.01); for sprints ≤ 15 m (ES = −0.55; 95% CI = −0.81, −0.29; Z = 4.15; *p* < 0.01); for sprints ≥ 20 m (ES = −0.56; 95% CI = −0.87, −0.24; Z = 3.49; *p* < 0.01); for repeated sprint ability (RSA) mean (ES = −0.33; 95% CI = −0.61, −0.05; Z = 2.34; *p* = 0.02); and for RSA % decrement (ES = −0.38; 95% CI = −0.74, −0.02; Z = 2.06; *p* = 0.04). However, most included studies were based on pre–post designs without a control group, and additional analyses with control groups showed smaller or non-significant effects. **Conclusions**: The results indicate that training programs incorporating methods such as strength training, plyometrics, and high-intensity interval training (HIIT) improve performance in vertical jump, short- and long-sprint speed, and RSA scores. These findings highlight the importance of developing evidence-based interventions to maximize physical performance in futsal players.

## 1. Introduction

Futsal is a team-based indoor sport, in which two teams of five players (four outfield players and a goalkeeper) compete against each other [1,2]. Futsal is currently one of the most widely played sports in the world, both at amateur and professional levels [3,4,5]. The game is played on a 40 m × 20 m court in two 20-min halves with a stopped clock. The objective is to score more goals than the opposing team [6,7]. It is a high-intensity sport that combines explosive movements such as acceleration, deceleration, sprinting, and changes of direction [3,8,9,10], requiring specific physical conditioning to enable players to sustain peak performance throughout the match [1,2,4]. At the competitive level, the sport’s physical and physiological demands have prompted researchers to investigate diverse training methods aimed at optimizing physical performance while minimizing the risk of injury [11,12,13,14], reflecting a continuous evolution in the scientific literature [15,16,17].

In recent years, systematic reviews on futsal have been conducted regarding the epidemiology of injuries [18,19,20], on injury prevention programs [13], anthropometric profiles of players [21], characteristics of futsal players [12], talent development in futsal players [4] and psychological studies applied to futsal [22].

In line with the strategies followed by other sports, such as football [23], numerous strength training programs have been explored to enhance futsal players’ physical and physiological capacities [24,25]. For example, plyometrics [25,26], high-intensity interval training (HIIT) [27] and combinations of these methods [28,29,30] have been used to improve specific strength, power, endurance, and agility in futsal [12,31]. However, the effectiveness of these programs varies depending on a number of factors such as program duration, intensity, frequency, and the competitive level of the players [32,33]. Thus, further research is required to understand the methods that best fit futsal requirements.

From a physiological and neuromuscular perspective, different training modalities induce specific adaptations that may explain their effects on performance. Strength training primarily enhances maximal force production and neuromuscular recruitment, which are key determinants of sprint and jump performance [34]. Plyometric training improves the efficiency of the stretch-shortening cycle, increasing the ability to rapidly generate force during explosive movements [35,36]. HIIT contributes to both aerobic and anaerobic adaptations, improving the capacity to sustain repeated high-intensity efforts, which is essential in futsal [37,38]. In addition, speed training enhances neuromuscular coordination and rate of force development, directly impacting acceleration and maximal sprint performance [39,40]. Understanding these underlying mechanisms is essential for optimizing training prescription and interpreting the effectiveness of different interventions.

Given the growing number of studies on training programs in futsal, conducting a systematic review and meta-analysis is essential to synthesize the existing evidence and provide a clearer understanding of effective training strategies. Although futsal research has been emerging and growing in recent years, it is still limited compared to other similar sports, where numerous meta-analyses have been conducted to investigate the impact of different training programs on physical performance [25,41,42,43,44,45,46]. To the best of our knowledge, no previous meta-analysis has specifically examined the effects of different training interventions on physical performance in futsal players. Therefore, conducting a meta-analysis is of great interest as it allows for the synthesis of existing evidence and helps fill an important gap in the scientific literature. This would not only help to identify the most effective training strategies for improving performance and minimizing the risk of injury but also to establish clear and useful benchmarks for professionals based on the studies conducted to date.

To understand the effects of different training programs in futsal, it is essential to use reliable and validated physical performance tests that ensure accurate assessment of both acute and chronic adaptations induced by training [47,48,49]. The application of standardized measurement protocols ensures consistency, comparability, and validity across studies, enabling a more robust understanding of training-induced changes. Rather than simply selecting assessment tools, it is crucial to establish protocols that minimize variability and ensure accurate monitoring of the effects of different interventions. Among the most commonly used physical performance tests in futsal are the countermovement jump (CMJ), speed tests covering different distances, and repeated sprint ability (RSA), which provide essential insights into key physical capabilities such as muscular strength, explosive power, movement efficiency, and intermittent effort performance, all of which are critical in competitive futsal [1,12,50]. Beyond assessing physical qualities, these tests should be systematically implemented to monitor individual progression and evaluate the effectiveness of training interventions.

Accordingly, the main objective of this systematic review and meta-analysis was to analyze the effects of training programs on different variables of physical performance in futsal players and their implications for player development. By identifying the methodological and design characteristics that lead to superior performance outcomes, this study aims to provide evidence-based recommendations for coaches and sports professionals to optimize training strategies.

## 2. Materials and Methods

### 2.1. Experimental Approach to the Problem

This systematic review and meta-analysis followed the Preferred Reporting Items for Systematic Reviews and Meta-Analyses statement (PRISMA) [51] and adheres to the ethical principles of the Declaration of Helsinki. The study was pre-registered in PROSPERO (CRD42024520512).

### 2.2. Literature Search

Two researchers (OVG and EMP) independently conducted a systematic literature search up to 31 October 2025 in the following electronic databases: PubMed, Web of Science, and SportDiscus. The search strategy was based on the PICOS approach (population, intervention, comparison, outcome, and study design) (Table 1) [51]. The selected search terms related to futsal, interventions and performance were combined using Boolean logic: (“futsal” OR “indoor soccer” OR “five-a-side soccer”) AND (“training” OR “intervention”) AND (“performance” OR “prevention” OR “strength” OR “power” OR “plyometric” OR “neuromuscular” OR “speed” OR “sprint” OR “HIIT” OR “change of direction” OR “agility”). In addition, gray literature and the reference lists of included studies were hand-searched to identify any additional potentially relevant articles.

### 2.3. Procedures

**Figure 1:**
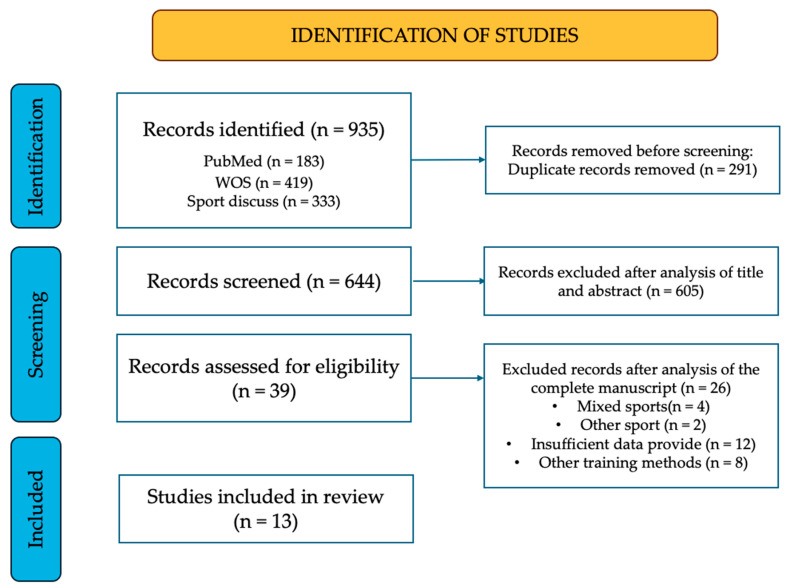
PRISMA flow chart for inclusion and exclusion of studies.

In order to include studies in the meta-analysis, a thorough review was carried out using a systematic process. First, the titles of relevant articles were assessed; then, the abstracts were analyzed, and finally, the full-text published articles were examined. Only peer-reviewed studies were included. The full search and selection process is shown in Figure 1. This process was carried out independently by two authors (OVG and EMP). Disagreements between the reviewers regarding study eligibility were resolved by consensus with a third author. Data extraction from the selected studies was also performed independently by two authors using a form designed in Microsoft Excel (Microsoft Corporation, Redmond, WA, USA).

The following criteria determined the eligibility of studies for inclusion in the review: Interventions had to have a minimum duration of 4 weeks and include training programs focused on strength, plyometrics, speed, or HIIT. The selected studies reported reliable measurable outcomes of players’ strength. In addition, only randomized controlled trials or quasi-experimental designs with comparable data were included.

Three primary tests were considered for data extraction: (i) vertical jump, (ii) linear sprint, and (iii) RSA test. After conducting a literature review, it was identified that these tests were the most frequently used in the analyzed studies, which led to their selection as the main variables. Vertical jump was generally assessed using the CMJ test. Linear sprint performance was recorded at different distances: For the acceleration phase, the time required to cover up to 15 m was used; for sprint performance, studies measuring the time to cover at least 20 m were included. RSA was analyzed using two variables: The Repeated Sprint Ability Mean (RSA mean) represents the average time recorded during a series of repeated sprints in a RSA test; the percentage Decrement in Repeated Sprint Ability (RSA %Dec) assesses the ability to maintain performance over successive sprint repetitions, reflecting accumulated fatigue during high-intensity intermittent exercise. A lower RSA %Dec value indicates better repeated sprint endurance, whereas a higher RSA %Dec value indicates greater loss of performance due to fatigue.

### 2.4. Statistical Analyses

Meta-analytic comparisons were performed using RevMan software version 5.3 [52]. A total of 13 studies comprising 23 experimental groups were included. Means and standard deviations (SD) from pre- and post-intervention assessments within the experimental groups were used to calculate effect sizes (ES). Effect sizes were adjusted using Hedges’ small sample size bias correction [53]. The random effects model with inverse variance (IV) weighting was used for the meta-analysis because it assigns proportional weights to studies based on the size of their individual standard errors [54] and accounts for heterogeneity among studies [55]. Effect sizes were represented by standardized mean differences (Hedges’ g) and reported with 95% confidence intervals (CI). The calculated ES values were interpreted based on the conventions for standardized mean differences described by Hopkins et al. [56] (<0.2 = trivial; 0.2–0.6 = small; 0.6–1.2 = moderate; 1.2–2.0 = large; 2.0–4.0 = very large; >4.0 = extremely large). The degree of heterogeneity among the included studies was assessed using the I^2^ statistic, which represents the proportion of the variability in effects that is due to heterogeneity rather than chance [51]. Low, moderate, and high levels of heterogeneity correspond to I^2^ values of 25%, 50%, and 75%, respectively, although these thresholds are considered tentative [57]. The χ^2^ (chi-squared) statistic was used to determine whether observed differences in outcomes were consistent with random variation alone. A low *p*-value, or a large χ^2^ statistic relative to the degrees of freedom, provides evidence of heterogeneity in intervention effects beyond what can be attributed to chance [54]. Given that the majority of included studies (8 out of 13) were based on pre–post designs without a control group, a secondary meta-analysis was conducted, including only studies with a control group (*n* = 5), to provide an additional estimate of the intervention effects based on controlled comparisons. In this analysis, effect sizes were calculated using standardized mean differences (SMD) based on post-intervention values, directly comparing the experimental and control groups. Subgroup analyses according to training modality were considered but not performed due to the limited number of studies per category and the heterogeneity of the interventions. Publication bias was not formally assessed because the small number of studies included in most outcome-specific analyses limited the interpretability and reliability of both funnel plots and formal asymmetry tests. Therefore, the possibility of publication bias cannot be excluded and should be considered when interpreting the findings. The Physiotherapy Evidence Database (PEDro) scale was used to assess the risk of bias and methodological quality of the eligible studies included in the meta-analysis. This scale assesses the internal validity of a study on a scale from 0 (high risk of bias) to 10 (low risk of bias) for each methodological item listed in Table 2. A score of ≥6 represents the threshold for studies with a low risk of bias [55].

## 3. Results

### 3.1. Study Selection

A total of 935 studies were collected in the identification phase. After removing duplicates, 644 publications were selected for screening. After reviewing the titles and abstracts, 605 articles were excluded. The remaining 39 records were further assessed using the defined inclusion and exclusion criteria, resulting in the rejection of 26 records. A total of 13 studies were included in the systematic review and meta-analysis (Figure 1) [58,59,60,61,62,63,64,65,66,67,68,69,70]. The interventions conducted by the author Torres-Torrelo et al. in their two studies are identical [66,67], but each article analyzes different variables.

### 3.2. Methodological Quality

The methodological quality of the studies included in the review was assessed using the PEDro scale, which comprises 11 dichotomous items (yes/no), although only 10 items are included in the final score because the first item relates to external validity (eligibility criteria). The reviewers, OVG and EMP, performed the assessment independently. Upon completion, both reviewers discussed the results and reached consensus on each item scored. A full and detailed description of the scores achieved by each study on this scale is given in Table 2. One study achieved a score of 8/10 [68], one study scored 7/10 [59], seven studies scored 6/10 [60,61,63,64,65,66,67], three studies scored 5/10 [58,69,70] and one study scored 4/10 [62].

**Table 2:** The Physiotherapy Evidence Database (PEDro) scale ratings.

Studies	1	2	3	4	5	6	7	8	9	10	11	T
Campos et al. (2021) [58]	1	0	0	1	0	0	0	1	1	1	1	5
Gómez et al. (2023) [59]	1	1	0	1	0	0	1	1	1	1	1	7
Iodice et al. (2020) [60]	1	1	0	1	0	0	0	1	1	1	1	6
Lago et al. (2018) [61]	1	1	0	1	0	0	0	1	1	1	1	6
Marques et al. (2022) [62]	1	0	0	0	0	0	0	1	1	1	1	4
Paz-Franco et al. (2017) [63]	1	1	0	1	0	0	0	1	1	1	1	6
Soares et al. (2014) [64]	1	1	0	1	0	0	0	1	1	1	1	6
Teixeira et al. (2019) [65]	1	1	0	1	0	0	0	1	1	1	1	6
Torres et al. (2017) [66]	1	1	0	1	0	0	0	1	1	1	1	6
Torres et al. (2018) [67]	1	1	0	1	0	0	0	1	1	1	1	6
Villanueva et al. (2024) [68]	1	1	0	1	1	0	1	1	1	1	1	8
Yanci et al. (2017) [69]	1	1	0	1	0	0	0	1	1	1	1	5
Zhai et al. (2024) [70]	1	0	0	1	0	0	0	1	1	1	1	5

### 3.3. Study Characteristics

The characteristics of the participants and programming parameters from the 13 studies incorporated in the meta-analysis are indicated in Table 3. A total of 23 trials with 297 participants were included in the meta-analysis (Table 4). It should be noted that the majority of included studies were based on pre–post designs without a control group, which should be considered when interpreting the results.

### 3.4. Main Effect

#### 3.4.1. Vertical Jump Performance

**Figure 2:**
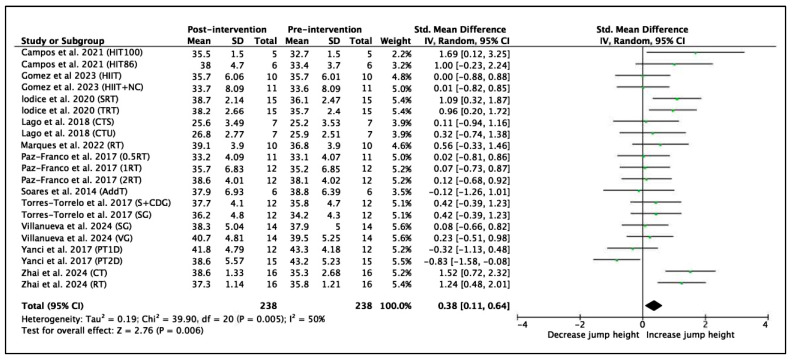
Forest plot of effect sizes for training programs with 95% confidence intervals (CI) on vertical jump performance (cm) [58,59,60,61,62,63,64,66,68,69,70]. IV: inverse variance method; SD: standard deviation; Std: standardized; df: degrees of freedom.

A total of 11 trials with 21 experimental groups were selected to analyze vertical jump performance, measured in centimeters. A total of 238 participants were included in the meta-analysis. The vertical jump variable was evaluated using the SMD using the IV method. The results show an SMD = 0.38 (95% CI = 0.11, 0.64), indicating a significant improvement in players’ performance after the interventions (Z = 2.76, *p* = 0.01). The heterogeneity between studies was moderate (I^2^ = 50%). The χ^2^ statistic was 39.90 with 20 degrees of freedom (*p* = 0.01), indicating that the variability between studies is statistically significant. These results are shown in Figure 2.

**Figure 3:**
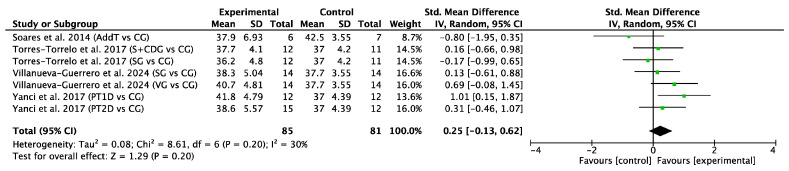
Forest plot of effect sizes for training interventions compared with control groups on countermovement jump (CMJ) performance [64,66,68,69]. (IV: inverse variance method; SD: standard deviation; CI: confidence interval).

A total of 7 comparisons from 5 studies were included to analyze CMJ performance using a control-group comparison. A total of 166 participants were included (85 in the experimental group and 81 in the control group). The CMJ variable was evaluated using the SMD with the IV method. The results showed an SMD of 0.25 (95% CI = −0.13, 0.62), indicating a small, non-significant improvement in CMJ performance in favor of the experimental group compared to the control group. The test for overall effect was not statistically significant (Z = 1.29, *p* = 0.20). Heterogeneity between studies was low to moderate (I^2^ = 30%). The χ^2^ test was not statistically significant (χ^2^ = 8.61, df = 6, *p* = 0.20), suggesting that the observed variability between studies is likely due to chance rather than true heterogeneity. These results are shown in Figure 3.

#### 3.4.2. Linear Sprint Time

**Figure 4:**
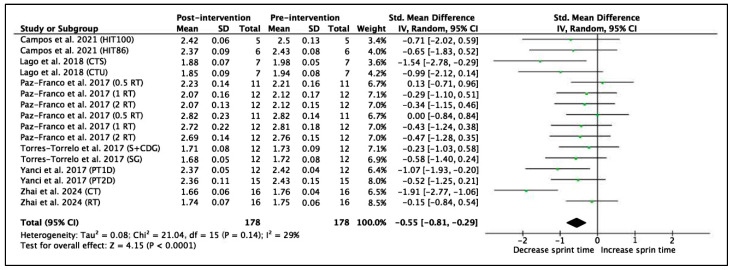
Forest plot of effect sizes for training programs with 95% confidence intervals (CI) on linear sprint acceleration performance (s) [58,61,63,66,69,70]. IV: inverse variance method; SD: standard deviation; Std: standardized; df: degrees of freedom. The interventions by Paz-Franco et al. (2017) [63] are repeated twice because times for both 10 m and 15 m were included.

Six studies with a total of 16 experimental groups were selected to analyze the acceleration phase of sprint performance at distances ≤ 15 m. A total of 178 participants were included in the meta-analysis. The acceleration phase was measured in time (seconds) in all studies. The meta-analysis shows an SMD = −0.55 (95% CI = −0.81, −0.29), indicating a significant improvement in short sprint time after the interventions (Z = 4.15, *p* < 0.0001). The heterogeneity between studies was moderate to low (I^2^ = 29%, *p* = 0.14). The χ^2^ statistic was 21.07 with 15 degrees of freedom (*p* = 0.14), indicating that the observed variability between studies is not statistically significant and is primarily due to chance. These results are shown in Figure 4.

**Figure 5:**
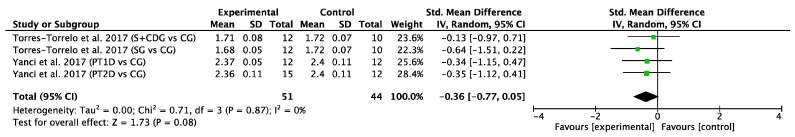
Forest plot of effect sizes for training programs with control groups and 95% confidence intervals (CI) on linear sprint acceleration performance (s) [66,69]. (IV: inverse variance method; SD: standard deviation; Std: standardized; df: degrees of freedom).

Four comparisons from two studies were included to analyze linear sprint acceleration performance at distances ≤ 15 m using a control-group comparison. A total of 95 participants were included (51 in the experimental group and 44 in the control group). The acceleration phase was measured in time (seconds) in all studies. The meta-analysis shows an SMD = −0.36 (95% CI = −0.77, 0.05), indicating a non-significant improvement in short sprint time in favor of the experimental group (Z = 1.73, *p* = 0.08). The heterogeneity between studies was null (I^2^ = 0%, *p* = 0.87). The χ^2^ statistic was 0.71 with 3 degrees of freedom (*p* = 0.87), indicating that the observed variability between studies is not statistically significant and is primarily due to chance. These results are shown in Figure 5.

**Figure 6:**
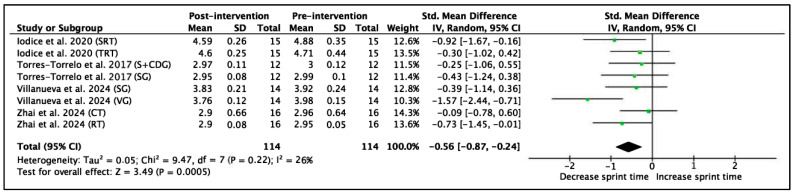
Forest plot of effect sizes for training programs with 95% confidence intervals (CI) on linear sprint performance (s) (≥20 m) [60,66,68,70]. IV: inverse variance method; SD: standard deviation; Std: standardized; df: degrees of freedom.

Four studies with a total of 8 experimental groups and 114 participants were included to analyze sprint performance at distances of 20 m or more. The variable was measured in time (seconds). The results of the meta-analysis show an SMD = −0.56 (95% CI = −0.87, −0.24), indicating a significant improvement in sprint time after the interventions (Z = 3.49, *p* = 0.0005). Heterogeneity among studies was low to moderate (I^2^ = 26%). The χ^2^ statistic was 9.47 with 7 degrees of freedom (*p* = 0.22), suggesting that the observed variability between studies was not statistically significant and was primarily due to chance. These results are shown in Figure 6.

**Figure 7:**
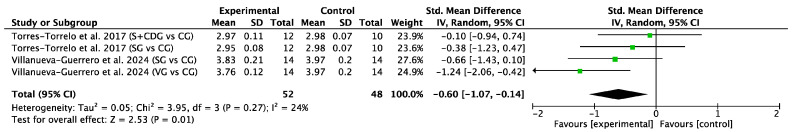
Forest plot of effect sizes for training programs with control groups and 95% confidence intervals (CI) on linear sprint performance (≥20 m) [66,68]. (IV: inverse variance method; SD: standard deviation; Std: standardized; df: degrees of freedom).

Four comparisons from two studies were included to analyze linear sprint performance at distances of 20 m or more using a control-group comparison. A total of 100 participants were included (52 in the experimental group and 48 in the control group). Sprint performance was measured in time (seconds) in all studies. The results of the meta-analysis show an SMD = −0.60 (95% CI = −1.07, −0.14), indicating a significant improvement in sprint time in favor of the experimental group compared to the control group (Z = 2.53, *p* = 0.01). Heterogeneity between studies was low (I^2^ = 24%). The χ^2^ statistic was 3.95 with 3 degrees of freedom (*p* = 0.27), suggesting that the observed variability between studies was not statistically significant and was primarily due to chance. These results are shown in Figure 7.

#### 3.4.3. RSA

**Figure 8:**
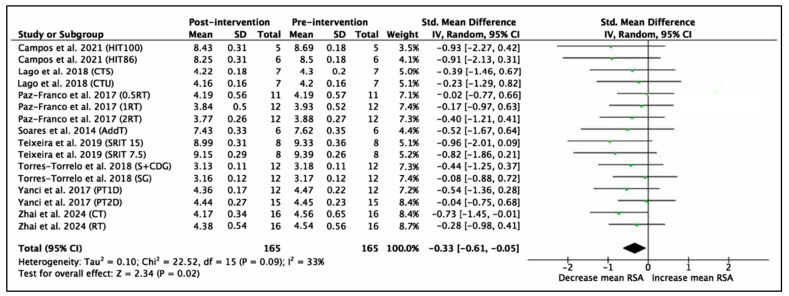
Forest plot of effect sizes for training programs with 95% confidence intervals (CI) on RSA mean performance (s) [58,61,63,64,65,67,69,70]. IV: inverse variance method; SD: standard deviation; Std: standardized; df: degrees of freedom.

Eight studies with a total of 16 experimental groups and 165 participants were included to analyze mean RSA performance. The variable was measured in time (seconds). The results of the meta-analysis show an SMD = −0.33 (95% CI = −0.61, −0.05), indicating a significant improvement in mean RSA performance after the interventions (Z = 2.34, *p* = 0.02). The heterogeneity between studies was moderate (I^2^ = 33%). The χ^2^ statistic was 22.52 with 15 degrees of freedom (*p* = 0.09), suggesting that the observed variability between studies was not statistically significant and could be primarily explained by chance. These results are shown in Figure 8.

**Figure 9:**
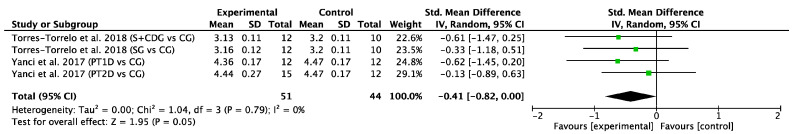
Forest plot of effect sizes for training programs with control groups and 95% confidence intervals (CI) on RSA mean performance (s) [67,69]. (IV: inverse variance method; SD: standard deviation; Std: standardized; df: degrees of freedom).

Four comparisons from two studies were included to analyze mean RSA performance using a control group comparison. A total of 95 participants were included (51 in the experimental group and 44 in the control group). The RSA mean variable was measured in time (seconds) in all studies. The results of the meta-analysis show an SMD = −0.41 (95% CI = −0.82, 0.00), indicating a small improvement in mean RSA performance in favor of the experimental group compared to the control group. The test for overall effect was at the threshold of statistical significance (Z = 1.95, *p* = 0.05). The heterogeneity between studies was null (I^2^ = 0%). The χ^2^ statistic was 1.04 with 3 degrees of freedom (*p* = 0.79), suggesting that the observed variability between studies was not statistically significant and was primarily due to chance. These results are shown in Figure 9.

**Figure 10:**
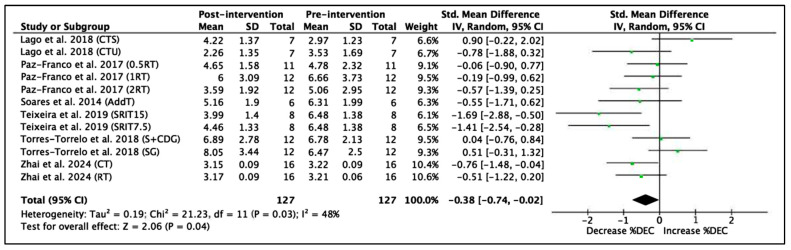
Forest plot of effect sizes for training programs with 95% confidence intervals (CI) on RSA %Dec performance [61,63,64,65,67,70]. IV: inverse variance method; SD: standard deviation; Std: standardized; df: degrees of freedom.

Six studies with a total of 12 experimental groups and 127 participants were included to analyze the RSA %Dec variable. The results of the meta-analysis show an SMD = −0.38 (95% CI = −0.74, −0.02), indicating a significant improvement in the ability to maintain performance during repeated sprints after the interventions (Z = 2.06, *p* = 0.04). The heterogeneity between studies was moderate, with I^2^ = 48%. The χ^2^ statistic was 21.23 with 11 degrees of freedom (*p* = 0.03), indicating that the heterogeneity between studies was statistically significant. These results are shown in Figure 10.

**Figure 11:**
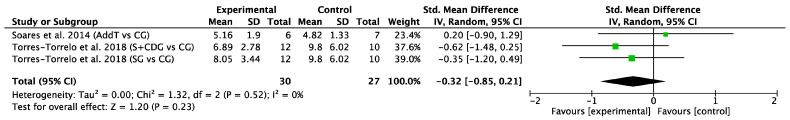
Forest plot of effect sizes for training programs with control groups and 95% confidence intervals (CI) on RSA %Dec performance [64,67]. (IV: inverse variance method; SD: standard deviation; Std: standardized; df: degrees of freedom).

Three comparisons from two studies were included to analyze RSA percentage decrement (RSA %Dec) using a control group comparison. A total of 57 participants were included (30 in the experimental group and 27 in the control group). The RSA %Dec variable was evaluated in all studies. The results of the meta-analysis show an SMD = −0.32 (95% CI = −0.85, 0.21), indicating a small, non-significant improvement in RSA %Dec in favor of the experimental group compared to the control group. The test for overall effect was not statistically significant (Z = 1.20, *p* = 0.23). The heterogeneity between studies was null (I^2^ = 0%). The χ^2^ statistic was 1.32 with 2 degrees of freedom (*p* = 0.52), suggesting that the observed variability between studies was not statistically significant and was primarily due to chance. These results are shown in Figure 11.

## 4. Discussion

The main findings of this meta-analysis indicate that training programs that focus on strength and speed improve vertical jump, linear sprint, and RSA performance in futsal players. This highlights the importance of incorporating strength, speed, and plyometric training programs into futsal-specific training routines in order to develop the physical capacities required in this sport. A key aspect to consider when interpreting the present findings is that the majority of included studies were based on pre–post designs without a control group. This methodological limitation makes it difficult to isolate the true effect of the intervention from other factors such as seasonal variations, accumulated fatigue, or natural adaptations to training. This issue is particularly relevant in futsal, where players are exposed to high training and competitive loads. Consequently, the reported effect sizes may be overestimated and should therefore be interpreted with caution. This limitation is partly explained by the limited number of intervention studies conducted in futsal to date. However, the growing interest in this research area suggests that future studies will likely adopt more robust methodological designs, including the use of control groups. Due to the limited number of studies and the heterogeneity of the interventions, subgroup analyses according to training modality were not performed. In several cases, the number of studies per category would have been insufficient to ensure statistical power and reliable estimates. Additionally, many interventions included combined or hybrid approaches (e.g., strength and plyometric training), making strict classification difficult and potentially arbitrary.

### 4.1. Vertical Jump Performance

The analysis of the effects of training programs on vertical jump performance in futsal showed significant results (Z = 2.76; *p* < 0.01). The studies included in the meta-analysis showed small but significant improvements (ES = 0.38) in vertical jump performance. According to the scientific literature, meta-analyses of the effects of interventions in diverse team sports (such as soccer, basketball, or volleyball) show similar results (ES = 0.53 to 1.09) [25,71,72]. No specific reviews have been carried out on training programs in futsal, which highlights the need for further studies to fill this knowledge gap. The main objectives of training programs are to improve physical performance and prevent injuries, which are fundamental pillars of player development. From a mechanistic perspective, these improvements in vertical jump performance may be explained by neuromuscular adaptations induced by the different training interventions applied, such as increased motor unit recruitment, enhanced rate of force development, and improvements in inter- and intramuscular coordination [35,73]. These adaptations are particularly relevant for explosive actions such as jumping, where the ability to rapidly generate force is a key futsal performance determinant [4,74,75].

The interventions that showed the best results in vertical jump performance were the HIIT100 training programs by Campos et al. [58] and the CT and RT programs by Zhai et al. [70], which achieved substantial improvements with a high magnitude (ES = 1.24 to 1.69). The interventions by Iodice et al. [60], with their SRT and TRT groups, showed moderate to substantial improvements (ES = 0.96 to 1.09), as did the HIIT86 training program by Campos et al. (ES = 1.00) [58]. In the shuttle-run HIIT programs studied by Campos et al. [58], an important difference was found in the intensity of the sessions. Participants in the HIIT100 program performed exercises at 100% intensity according to the FIET test, whereas in the HIIT86 program, the intensity was reduced to 86%. This higher intensity in the HIIT100 program resulted in greater improvements in vertical jump performance compared with HIIT86. This may be explained by the greater neuromuscular and metabolic stimulus associated with higher-intensity efforts, suggesting that training intensity may be a key factor influencing the magnitude of performance adaptations. Higher-intensity stimuli can enhance both muscle activation and the capacity to tolerate high levels of mechanical load, thereby contributing to improved explosive performance.

In addition, the data from the forest plot (Figure 2) suggest that studies with high-intensity training programs lasting longer than 8 weeks tend to achieve larger effect sizes, supporting the idea that these factors are key determinants in improving vertical jump performance. This finding is consistent with the meta-analysis conducted by Pardos-Mainer et al. [25], who demonstrated that durations of 8 weeks or more increased the effectiveness of vertical jump performance in female soccer players. Moreover, these results suggest that not only the intensity but also the sustained duration of the training program may be a key factor for achieving long-term performance improvements. Longer training durations likely allow for the accumulation of both neural and structural adaptations, including improvements in muscle–tendon stiffness and neuromuscular efficiency, which are essential for maximizing force production during vertical jump tasks [76].

In contrast, the training programs of Yanci et al. [69] and Soares et al. [64] had negative effects on vertical jump performance (ES = −0.83 to −0.12). The training programs of Yanci et al. [69] included specific plyometric sessions with exercises such as CMJ, Drop Jump (DJ), HJ, and Lateral Jump (LJ), both bilateral and unilateral, over a period of 6 weeks. Soares et al. [64] included additional sprint training three times a week in Brazilian first division futsal players and showed negative results on vertical jump performance. A possible explanation for the decrease in CMJ performance observed in these studies could be the timing of the final evaluation, which coincided with the final phase of the season, a period associated with high levels of accumulated fatigue [77]. In this context, fatigue-related reductions in neuromuscular function may impair the ability to produce force rapidly, negatively affecting jump performance despite ongoing training stimuli [78]. This is consistent with the results observed in Table 1, where studies with shorter durations (<6 weeks) or those focusing exclusively on specific exercises, such as Soares et al. [64], showed less efficacy than longer-duration combined protocols. This finding contrasts with the existing literature, where meta-analyses to date indicate that plyometric training typically leads to significant improvements in vertical jump performance [79]. These results highlight the importance of carefully planning the timing of assessments and training sessions to avoid interfering with the accumulated loads during the competition season.

The main strength exercises used in the training programs analyzed included the squat, which was present in the programs of Marques et al. [62], Torres-Torrelo et al. [66], Zhai et al. [70], as well as the Nordic Curl in the SG programs by Villanueva et al. [68], the HIIT + NC program by Gómez et al. [59] and the program by Marques et al. [62]. Table 1 shows that programs that combined strength exercises with speed or plyometric components had larger effect sizes than those that focused on only one type of exercise [32]. These exercises have been shown to be effective not only in improving vertical jump performance but also in preventing injury [80,81]. The primary goal of strength training is to develop maximal strength to optimize physical performance but combining it with maximal speed tasks and plyometrics enhances these benefits [32]. This combined approach likely maximizes performance adaptations by simultaneously targeting force production, stretch–shortening cycle efficiency, and movement velocity, all of which are key contributors to vertical jump performance. Specific speed programs, such as those of Campos et al. [58] and the VG training program of Villanueva et al. [68], also stood out, especially when they lasted longer than 8 weeks. This suggests that programs of longer duration provide a more sustained and consistent stimulus, leading to significant adaptations in vertical jump performance.

Evidence suggests that it is beneficial for futsal players to implement training programs that last longer than 8 weeks. Incorporating basic strength exercises, such as squats, and combining strength training with maximal-speed and plyometric tasks is beneficial for improving vertical jump performance in futsal players.

### 4.2. Linear Sprint Time

The present meta-analysis shows significant improvements in linear sprint performance in both the acceleration phase (≤15 m) and the maximal speed phase (≥20 m), with significant effects (Z = 4.15, *p* < 0.01; Z = 3.49, *p* < 0.01) and small effect sizes (ES = −0.55 and −0.56). These results are consistent with previous systematic reviews and meta-analyses that reported similar effect sizes (ES = −0.66 to −0.40) [25,82,83], strengthening the robustness of the interventions. Such improvements in sprint performance may be explained by neuromuscular adaptations induced by the training interventions, including increased rate of force development, enhanced intermuscular coordination, and improved ability to apply force in the horizontal direction, all of which are critical determinants of sprint acceleration and maximal speed [84,85].

The interventions with the greatest effect on the acceleration phase (≤15 m) were the CTS training programs by Lago et al. [61] and the CT program by Zhai et al. [70], characterized by specific strength exercises and strength training on stable surfaces (ES = −1.91 to −1.54). Developing reactive power through exercises that integrate lower-body and core stability can optimize performance in the first meters, where horizontal power is key, crucial in futsal. This is particularly relevant during the initial phase of sprinting, where performance largely depends on the ability to generate high levels of horizontal force in a short time frame [84]. In addition, the PT1D intervention by Yanci et al. [69] and the programs by Campos et al. [58] showed a moderate effect (ES = −0.65 to −1.07). Furthermore, the inclusion of plyometric tasks by Yanci et al. [69] and Zhai et al. [70] is directly related to improvements in muscle elasticity and the use of the stretch-shortening cycle (SSC), which enhances the efficiency of the stretch–shortening cycle and the ability to rapidly produce force [86]. The muscular actions of the SSC mimic those produced during sprint acceleration [87]. In sports such as futsal and football, where explosive actions often last between 2 and 4 s, these interventions have a direct impact on competitive performance [88,89]. The ability to accelerate quickly within ≤15 m is essential as it reflects the real demands of the game and helps to overtake opponents.

On the other hand, the intervention that showed the best results in sprint performance (≥20 m) with a large magnitude (ES = −1.57) was the VG training program by Villanueva et al. [68]. This intervention included specific speed exercises, incorporating sled-pulling tasks that have been confirmed to be effective in improving sprint performance [90]. These improvements may be attributed to enhanced neuromuscular coordination and force application at higher velocities, which are essential for maintaining stride length and frequency during maximal sprinting. Additionally, general and specific strength training in the SRT interventions by Iodice et al. [60] and the RT program by Zhai et al. [70] showed a moderate effect (ES = −0.92 to −0.73) on sprint performance (≥20 m). Strength training not only facilitates the development of the required musculature but also promotes neuromuscular adaptations aligned with the specific demands of sprinting [91,92,93]. In particular, increases in maximal strength can enhance the ability to produce force rapidly, thereby improving both acceleration and the transition to maximal speed. Furthermore, a recent study by Oleksy et al. highlights that general strength training should be complemented with specific exercises that meet the demands of sprints [94].

With regard to the linear sprint variable, it is suggested that the incorporation of specific speed tasks can improve sprint times in futsal players. In addition, strength training and plyometric work play a fundamental role in this process, as both increase the explosive capacity of the musculature and directly contribute to improving performance over the first few meters. This combined approach likely optimizes sprint performance by targeting both force production capacity and movement velocity, which are key components of sprint mechanics. Coaches should therefore design training programs based on an analysis of the demands of the sport and the characteristics of the athlete. Players with deficits in acceleration could benefit from plyometric exercises, while those who need to improve their maximum speed should prioritize sled drags [90] and sport-specific strength training.

### 4.3. RSA

The analysis of the effects of training programs on RSA in futsal shows significant results for both mean RSA (Z = 2.34; *p* = 0.02) and RSA %Dec (Z = 2.06; *p* = 0.04). The studies included in the meta-analysis show small but significant improvements in RSA mean (ES = −0.38) and RSA %Dec (ES = −0.38). These improvements are consistent with previous findings in sports with similar characteristics, such as football and other sports (ES = −0.34) [27,95]. However, other studies have not shown significant improvements in RSA with small-sided games or HIIT-based training (*p* = 0.18) [96], highlighting the importance of selecting appropriate training methods. From a physiological standpoint, improvements in RSA performance may be explained by both metabolic and neuromuscular adaptations induced by training, including enhanced phosphocreatine resynthesis, improved buffering capacity, and a greater ability to maintain force production during repeated high-intensity efforts [95,97].

The most effective programs, such as those by Campos et al. [58], Teixeira et al. [65] and the CT intervention by Zhai et al. [70] achieved significant improvements with moderate magnitudes (ES = −0.96 to −0.73) in the mean RSA variable. These programs share key characteristics, such as the inclusion of high-intensity speed tasks with short recovery periods, mimicking the intermittent demands typical of futsal. Training specificity emerges as a key principle for optimizing RSA capacity in real game contexts. In particular, the studies by Campos et al. [58] and Teixeira et al. [65] designed interventions that closely reflected the demands of the RSA test and the actions observed during a futsal match. This type of training stimulus likely enhances the ability to repeatedly produce high-intensity efforts by improving both energy system efficiency and neuromuscular resilience under fatigue conditions. On the other hand, the CT intervention by Zhai et al. [70] complemented this approach by combining basic strength exercises, such as high-intensity squats and deadlifts, with plyometric tasks, highlighting the importance of integrating different training modalities. Such combined approaches may further support RSA performance by simultaneously improving force production capacity and the efficiency of the stretch–shortening cycle under repeated effort conditions.

Regarding RSA %Dec, the most significant improvements were observed in the programs by Teixeira et al. [65] with a large magnitude (ES = −1.69 to −1.42), and a moderate magnitude in the CTU training programs by Lago et al. [61] and the CT program by Zhai et al. [70]. However, some studies, such as those by Torres-Torrelo et al. [67] and the CRS program by Lago et al. [61], showed negative effects on RSA %Dec, probably due to insufficient intensities or lack of specificity in the tasks proposed. This highlights the need to adapt training loads to maximize adaptations. Insufficient intensity or inadequate recovery periods may limit the development of metabolic adaptations required to sustain repeated efforts, thereby reducing improvements in fatigue resistance. In the case of core training analyzed by Lago et al. [61], it was observed that the use of unstable platforms led to moderate improvements (ES = −0.78), whereas the use of stable platforms reduced performance (ES = 0.90). These results highlight the importance of contextualizing core training in relation to the specific demands of the sport. A study comparing core training with speed training found no differences in RSA capacity between the training groups [98]. Huxel-Bliven et al. [99] emphasizes the importance of core training in improving athletic performance and preventing injuries [99], but further studies are needed to conclusively determine the effects of core training in different sports, particularly futsal.

RSA %Dec is a critical variable for assessing fatigue resistance during high-intensity intermittent efforts, a fundamental aspect of futsal [1,12]. In this context, the programs of Teixeira et al. [65] achieved significant improvements in RSA %Dec with an ES = −0.82 to −0.96. In addition, the training programs of Campos et al. [58] also showed moderately substantial effects on mean RSA (ES = −0.91 to −0.93). These programs are characterized by their specific design, which includes periods of exertion and recovery that simulate the most common actions in futsal, such as two consecutive sprints followed by 15 s of recovery [88]. Such intermittent structures are likely to optimize both aerobic and anaerobic energy system contributions, enhancing the ability to recover between efforts and sustain repeated sprint performance. The principle of training specificity states that adaptations are specific to the type of stimulus applied [100,101]. For example, Pyne et al. [102] found that RSA capacity was more closely related to short sprint qualities than to aerobic endurance, while Gerard et al. [103] emphasized the importance of replicating sprints under fatiguing conditions. Therefore, RSA training programs should prioritize exercises that mimic the real demands of futsal, not only in terms of intensity but also in terms of the temporal structure of efforts and recoveries. This aspect is particularly relevant given that many of the RSA protocols commonly used in the literature originate from other sports with different activity profiles. Consequently, adapting RSA assessments to better reflect the specific effort–recovery patterns of futsal may provide a more accurate evaluation of players’ performance.

In general, the literature suggests that incorporating training programs into futsal improves RSA capacity, as all training programs had positive effects on the mean RSA variable. Due to the heterogeneity of the studies conducted on futsal interventions to date, further research is needed to draw conclusive results. Coaches should design programs that include sport-specific intermittent tasks, such as speed circuits combined with plyometric exercises and core training on unstable surfaces, to improve both repeated sprint performance and fatigue resistance. This integrated approach likely enhances RSA by combining neuromuscular and metabolic adaptations, which are essential for maintaining performance during repeated high-intensity actions. In addition, the integration of match simulations with repeated sprints under fatiguing conditions could improve transfer to actual match play. Periodic assessments of RSA are also recommended to monitor the impact of interventions and to adjust training loads based on individual player needs. Finally, the combination of explosive strength training, plyometrics, and specific sprint tasks represents a comprehensive strategy to maximize performance in futsal and minimize the risk of injury.

### 4.4. Limitations

One of the main limitations of this meta-analysis is that most of the included studies did not include a control group and relied on pre–post designs; only 5 out of 13 articles included a control group [64,66,67,68,69]. This methodological limitation reduces the internal validity of the findings and makes it difficult to isolate the true effect of the intervention from other factors, such as seasonal variations, accumulated fatigue, or natural adaptations to training. As a result, the magnitude of the reported effects may be overestimated, and the findings should therefore be interpreted with caution. This limitation highlights the need for more robust methodological designs, particularly randomized controlled trials, in future studies. In addition, the relatively small number of studies included in this meta-analysis may limit the generalizability and robustness of the findings. Furthermore, the heterogeneity of the training interventions included in this meta-analysis limited the possibility of conducting subgroup analyses according to training modality. Similarly, the limited number of studies also precluded the use of meta-regression analyses. Another limitation relates to the variability in testing protocols used across studies (e.g., differences in sprint distances, RSA protocols, and jump assessments), which may affect the comparability of results. Additionally, the literature search was restricted to three databases (PubMed, Web of Science, and SPORTDiscus). Although these databases provide extensive coverage of sports science and performance-related research, the exclusion of other databases may have resulted in the omission of potentially relevant studies. However, to minimize this limitation, gray literature and the reference lists of included articles were also hand-searched to identify additional potentially relevant studies. Another limitation of this meta-analysis is that publication bias could not be formally assessed. The relatively small number of studies included in most outcome-specific analyses limited the interpretability and reliability of both funnel plots and formal statistical tests for asymmetry. Therefore, the potential influence of publication bias cannot be ruled out and should be considered when interpreting the present findings. Another relevant limitation is that the complexity of recruiting samples in futsal, particularly for the implementation of training programs during competitive seasons, must be acknowledged. Challenges include the limited availability of players, time constraints due to competitive commitments, and the need to ensure that participants strictly adhere to the criteria and protocols established in the training programs. These difficulties highlight the importance of carefully planning interventions and seeking solutions to improve feasibility and adherence in future studies.

As future lines of research, it would be valuable to establish a protocol for evaluating physical performance based on the demands of each sport. While it is common to find reference tests, such as the CMJ variable, it is necessary to standardize the distances for maximal linear sprint and RSA tests. In maximal linear sprint tests, distances range from 5 m to 60 m. For RSA tests, protocols include 6 to 9 repetitions with distances of 20 m to 25 m and recovery times of 20 to 25 s between repetitions. Standardizing these protocols would facilitate their practical application by physical trainers, improve the comparability of results, and optimize the design of sport-specific training programs for futsal. Additionally, future studies should further explore the implementation of more robust methodological designs, particularly randomized controlled trials with appropriate control groups, to provide stronger evidence on the effectiveness of training interventions in futsal.

### 4.5. Practical Applications

The findings of this meta-analysis provide relevant practical implications for coaches and strength and conditioning practitioners working in futsal. Training programs that combine strength, plyometric, and speed-oriented exercises appear to be the most effective for improving key performance variables such as vertical jump, linear sprint, and RSA. In particular, interventions lasting at least 8 weeks and performed at high intensity seem to produce greater improvements, especially in vertical jump performance. To enhance sprint performance, especially during the acceleration phase (≤15 m), practitioners should prioritize exercises that develop horizontal force production, including resisted sprinting and plyometric training. For maximal sprint performance (≥20 m), specific speed training should be complemented with strength training to optimize neuromuscular adaptations. Regarding RSA, training programs should include high-intensity intermittent efforts with short recovery periods that replicate the temporal structure of futsal match demands. These approaches are likely to improve both metabolic and neuromuscular capacity to sustain repeated high-intensity actions. Finally, coaches are encouraged to individualize training programs based on player characteristics, competitive demands, and the specific phase of the season, as training objectives and responses may vary depending on factors such as accumulated fatigue, match congestion, and performance priorities. Ensuring that training content closely reflects the physical and temporal requirements of futsal across the season may further enhance performance outcomes.

## 5. Conclusions

This meta-analysis shows that specific training programs have a significant effect on improving the physical performance of futsal players across key variables such as vertical jump (CMJ), sprint speed, and RSA. In particular, training programs that integrate strength, plyometric, and speed-oriented exercises appear to produce the most consistent improvements across these performance variables. Programs that combine strength, speed, and plyometric exercises over a period of 8 weeks or more produce greater effects on vertical jump performance. These findings suggest that both training content and duration are critical factors for maximizing neuromuscular adaptations in futsal players. On the other hand, specific speed training programs are most effective for improving linear sprint speed, especially during the acceleration phase, which is a key determinant of performance in futsal-specific actions. Similarly, high-intensity training with short recovery periods has the greatest effect on improving RSA capacity, likely due to its ability to replicate the intermittent nature of match play and to enhance both metabolic and neuromuscular responses to repeated efforts.

In addition, the results highlight the importance of designing training programs that are tailored to the specific characteristics of futsal, taking into account factors such as intensity, frequency, and duration of training. Coaches and practitioners should prioritize training strategies that reflect the temporal and physical demands of futsal, including short-duration high-intensity efforts combined with incomplete recovery periods. Although the training programs evaluated showed benefits, the variability in the methodological designs of the included studies reflects the need for standardization of interventions and evaluation parameters. However, these findings should be interpreted with caution due to the predominance of pre–post study designs without control groups. Future research should prioritize randomized controlled trials with appropriate control groups to provide more robust evidence on the effectiveness of training interventions in futsal.

## Figures and Tables

**Table 1:** Inclusion and exclusion criteria.

Category	Inclusion Criteria	Exclusion Criteria
Population	Male and female futsal players (junior and senior categories) aged 16 years or older.	Players under 16 years
Intervention/ Exposure	Strength, speed, plyometric, or high-intensity interval training (HIIT) programs.	Interventions without specific strength, speed, plyometric, or HIIT methods.
Comparison	Active control group, experimental group, or intra-group observations (pre- and post-intervention comparisons).	Absence of clear comparative data (neither between groups nor within the same group across different time points).
Outcome	At least one measure of physical performance (vertical jump, linear sprint, or repeated sprint ability) before and after the intervention.	Lack of baseline and/or follow-up data, or analysis of variables unrelated to physical performance.
Study design	Randomized controlled trials, quasi-experimental designs, or longitudinal observational studies with comparative data.	Cross-sectional studies, non-original reviews, or studies without clear comparative data.
Other	Only original and full-text studies published in English or Spanish	Studies not written in English or Spanish; non-original articles (reviews, letters to the editor, protocols, editorials, etc.).

**Table 3:** Characteristics of study participants.

Study	*N*	Group	Age	G	Level	Time	Type of Training	Test
Campos et al. (2021) [58]	11	HIIT86 (*n* = 6) HIIT100 (*n* = 5)	18.0 ± 1.1	M	Elite	Fr = 1 W = 10	Shuttle run HIIT 86% vs. Shuttle run HIIT 100%	FIET, treadmill incremental test, Sprint 15 m, CMJ, SJ, and HRV.
Gómez et al. (2023) [59]	21	HIIT (*n* = 10) HIIT + NC (*n* = 11)	20.9 ± 1.2	M	Amateur	Fr = 1 W = 4	HIIT vs. HIIT + NC	BMI, 30-15IFT, CMJ, ABK, Maximal isometric strength, and isometric H/Q ratio.
Iodice et al. (2020) [60]	30	SRT (*n* = 15) TRT (*n* = 15)	24.0 ± 1.5	M	Elite	Fr = 2 W = 8	SRT vs. TRT	CMJ, Isokinetic assessment, MVC, Sprint 30 m, and Sprint 60 m
Lago et al. (2018) [61]	14	CTS (*n* = 7) CTU (*n* = 7)	23.7 ± 5.1	F	Elite	Fr = 3 W = 6	Core strength training performed on stable (CTS) and unstable (CTU) surfaces	FMS, CMJ, Sprint 10 m and RSA
Marques et al. (2022) [62]	10	RT (*n* = 10)	24.8 ± 5.4	M	Elite	Fr = 2 W = 8	Resistance training	CMJ, IHAS, 1RM squat, and PP squat
Paz-Franco et al. (2017) [63]	35	0.5 RT (*n* = 11) 1 RT (*n* = 12) 2 RT (*n* = 12)	23.8 ± 5.2	M	Elite	Fr = 0.5/1/2 W = 6	3 groups Resistance training	CMJ, ABK, Sprint 5-10-15 m and RSA
Soares et al. (2014) [64]	13	AddT (*n* = 6) CG (*n* = 7)	21.4 ± 5.5	M	Elite	Fr = 3 W = 4	Sprint Training vs. CG	CMJ, SJ, YoYo, and RSA
Teixeira et al. (2019) [65]	16	SRIT7.5 (*n* = 7) SRIT15 (*n* = 9)	15.8 ± 0.2	F	Elite	Fr = 2 W = 5	2 groups Shuttle-run interval training (SRIT)	ITT, FIET and RSA
Torres et al. (2017) [66]	34	SG (*n* = 12) S + CDG (*n* = 12) CG (*n* = 10)	23.7 ± 4.1	M	Amateur	Fr = 2 W = 6	CG + Full squat exercise (SG) or full squat combined with change of direction exercises.	CMJ, Sprint 20 m, V-CUT, 1RM squat, and RSA
Torres et al. (2018) [67]	34	SG (*n* = 12) S + CDG (*n* = 12) CG (*n* = 10)	23.7 ± 4.1	M	Amateur	Fr = 2 W = 6	CG + Full squat exercise (SG) or full squat combined with change of direction exercises.	HJ, RSA, and 1RM full squat
Villanueva et al. (2024) [68]	42	SG (*n* = 14) VG (*n* = 14) CG (*n* = 14)	17.4 ± 0.6	M	Elite	Fr = 1 W = 8	SG vs. VG vs. CG	CMJ, HJ, Sprint 25 m, RSA, and VCUT
Yanci et al. (2017) [69]	39	PT1D (*n* = 12) PT2D (*n* = 15) CG (*n* = 12)	22.5 ± 5.0	M	Amateur	Fr =1/2 W = 6	Plyometric training	Sprint 15 m, CMJ, 505, RSA, and HJ
Zhai et al. (2024) [70]	32	CT (*n* = 16) RT (*n* = 16)	18.9 ± 0.8	M	Amateur	Fr = 2 W = 8	Complex training vs. resistance training	FSPT, RSA, Sprint 10 m, Sprint 20 m, 1RM squat, and CMJ

*N*: Number of participants; G: Gender; M: Male; F: Female; Fr: Frequency per week; W: Number of weeks; CG: Control Group; HIIT: High-intensity interval training; HIIT86: High-intensity interval training at 86% of the FIET test; HIIT100: High-intensity interval training at 100% of the FIET test; NC: Nordic Curl; SRT: Slow-speed resistance training with low intensity; TRT: Traditional resistance training; CTS: Core Strength Training using a stable surface; CTU: Core Strength Training using an unstable surface; RT: Resistance Training; AddT: Additional Sprint Training; SRIT: Shuttle-run interval training; SG: Squat Group; S + CDG: Squat Group with loaded change of direction; VG: Velocity Group; PT1D: Plyometric training 1 day per week; PT2D: Plyometric training 2 days per week; CT: Complex Training; FIET: Futsal Intermittent Endurance Test; CMJ: Countermovement Jump; SJ: Squat Jump; HRV: Heart Rate Variability; BMI: Body Mass Index; ABK: Countermovement Jump with Arm Impulse; MVC: Maximal Isometric Voluntary Contraction; FMS: Functional Movement Screen; RSA: Repeated Sprint Ability; IHAS: Isometric Hip Adduction Strength; 1RM: One-Repetition Maximum; PP: Peak Power; HJ: Horizontal Jump; FSPT: Futsal Special Performance Test.

**Table 4:** Description of training programs.

Training Program	*N*	Frequency	Time	Type of Training
Shuttle run HIIT 86% (HIIT86) [58]	6	1 per week	10 weeks	4 sets of 4 min bouts at 86% of PSFIET, with 3 min of passive recovery between sets. Each set included 8 repetitions of 15 s runs followed by 15 s of rest, with a change of direction every 3.75 s. A total of 128 accelerations and 96 decelerations were performed per session.
Shuttle run HIIT 100% (HIIT100) [58]	5	1 per week	10 weeks	8 sets of 60 s bouts at 100% of PSFIET, with 45 s of passive recovery between sets. Each set included 2 repetitions of 15 s runs followed by 15 s of rest, with a change of direction every 3.75 s. A total of 64 accelerations and 48 decelerations were performed per session.
HIIT [59]	10	1 per week	4 weeks	25 min HIIT circuit (3 rounds, 40 s work/15 s rest) with squats + press, planks + push-ups, wall squat + press, sprints, lateral plank, jumping jacks, lumbar rotations, and coordination drills
HIIT + NC [59]	11	1 per week	4 weeks	25 min HIIT circuit (3 rounds, 40 s work/15 s rest) with squats + press, planks + push-ups, wall squat + press, sprints, lateral plank, jumping jacks, lumbar rotations, and coordination drills + Nordic curl (10 min, 3 × 10 reps).
Slow-speed resistance training (SRT) [60]	15	2 per week	8 weeks	3 sets to failure at 50% 1RM on leg extension and leg curl isotonic machines, with tempo 3/0/3/0 and 60 s rest. Muscle time under tension was monitored per session.
Traditional resistance training (TRT) [60]	15	2 per week	8 weeks	6 sets of 8 reps at 80% 1RM on leg extension and leg curl isotonic machines, with tempo 1/0/1/0 and 60 s rest. Additional volume performed to match the muscle time under tension of the SRT group.
Core strength training performed on a stable surface (CTS) [61]	7	3 per week	6 weeks	Core-strengthening training on a stable surface for 6 weeks (3 sessions/week, ~20 min). The program included shoulder bridge, side plank, prone plank, and crunch, progressing every two weeks by increasing duration (30–50 s) and adding variations such as arm and leg lifts and the use of a futsal ball.
Core strength training performed on unstable surfaces (CTU) [61]	7	3 per week	6 weeks	Core-strengthening training on an unstable surface (Togu^®^ Dyn-Air) for 6 weeks (3 sessions/week, ~20 min). The same exercises as in CTS were performed, but on an unstable base to enhance neuromuscular activation and postural control, with progression in duration (30–50 s) and similar variations.
Resistance training (RT) [62]	10	2 per week	8 weeks	60 min with Smith Machine squats (2–3 sets, 3–6 reps, 45–65% 1RM, fast concentric, 2–3 min rest). Complemented with CMJ, Nordic curl, Copenhagen adductor, glute bridge, and core exercises with 30–60 s rest.
Resistance training (RT) [63]	11	1 per two weeks	6 weeks	Strength training with half squat on the Smith Machine, leg press, and hamstring curl, performed before field training. 1RM was used to set loads, with fast concentric and controlled eccentric phase (2–3 s). Load was adjusted based on OMNI-Res Scale RPE, increasing 5–15% if RPE < 9.
	12	1 per week		
	12	2 per week		
Additional sprint training (AddT) [64]	6	3 per week	4 weeks	2 sets of 6–8 × 30 m sprints with 20 s rest between sprints and 5 min between sets, increasing the number of sprints each week.
Shuttle-run interval training (SRIT7.5) [65]	7	2 per week	5 weeks	Shuttle-run interval training (4 sets of 4 min, 3 min rest) with 7.5 s running and 7.5 s pause at 89% PSFIET, with COD every 3.75 s. Load was progressively adjusted by increasing the distance if HR mean < 90% HRmax
Shuttle-run interval training (SRIT15) [65]	9	2 per week	5 weeks	Shuttle-run interval training (4 sets of 4 min, 3 min rest) with 15 s running and 15 s pause at 86% PSFIET, with COD every 3.75 s (3 direction changes per repetition). Load was progressively adjusted by increasing the distance if HR mean < 90% HRmax
Full squat exercise (SG) [66,67]	12	2 per week	6 weeks	Free-weight full squat program, with 2–3 sets of 4–6 reps, progressing movement velocity from 1.20 m/s (~45% 1RM) to 1.00 m/s (~58% 1RM) and 3 min rest between sets
Full squat combined with COD (S + CDG) [66,67]	12	2 per week	6 weeks	Combined free-weight full squat program, with 2–3 sets of 4–6 reps, progressing movement velocity from 1.20 m/s (~45% 1RM) to 1.00 m/s (~58% 1RM), with 2–5 sets of 10 s of COD drills in an 8 m three-square area, using 5–10 kg additional load depending on 1RM, with 2 min rest between COD sets and 3 min rest for squats.
Strength training (ST) [68]	14	2 per week	8 weeks	Tri-sets with eccentric, plyometric, and lumbo-pelvic strengthening exercises. Included eccentric quadriceps on the Russian belt, Nordic hamstring curl, front lunge with resistance band, jumps (vertical, horizontal, lateral, L-shaped), and planks (front, Copenhagen lateral), plus hip abduction/adduction resisted by a partner.
Velocity training (VG) [68]	14	2 per week	8 weeks	Tri-sets with linear speed load, COD drills, resisted accelerations, and lumbo-pelvic strengthening. Included 25 m sprint with parachute, 6 m acceleration with 7 kg ballast (10% body weight), 25 m sprint with 4 COD, plus band-resisted starts (frontal, cross, lateral), planks (front, Copenhagen lateral), and hip abduction/adduction resisted by a partner.
Plyometric training (PT) [69]	12	1 per week	6 weeks	Included horizontal and vertical jump exercises, unilateral and bilateral jumps, and change of direction drills. Sessions consisted of arm swing countermovement jumps, horizontal and vertical single-leg jumps, drop jumps (dominant and non-dominant leg), and side-to-side ankle hops. Exercises were performed for 3 sets with a progressive number of repetitions over six weeks
	15	2 per week		
Complex training (CT) [70]	16	2 per week	8 weeks	Combined resistance and plyometric exercises, structured in paired sets. Participants performed Squats or Deadlifts (4–6 reps) followed by Squat Jumps or High Pulls (10–12 reps) with 4 min rest intervals. The intervention lasted 8 weeks, with 1RM assessments every 4 weeks to adjust intensity
Resistance training (RT) [70]	16	2 per week	8 weeks	Squats or deadlifts can be combined with resistance training. Each session consisted of six sets (6–10 reps per set) with a 4 min rest between sets. The 8-week program included 1RM assessments every 4 weeks for intensity adjustments.

*N*: Number of participants; RM: Repetition Maximum.

## Data Availability

No new data were created or analyzed in this study. Data sharing is not applicable to this article.

## Abbreviations

The following abbreviations are used in this manuscript:

AddT	Additional Sprint Training
CMJ	Countermovement Jump
COD	Change of Direction
CT	Complex Training
CTS	Core Strength Training Performed on Stable Surfaces
CTU	Core Strength Training Performed on Unstable Surfaces
DJ	Drop Jump
ES	Effect Size
HIIT	High-Intensity Interval Training
HJ	Horizontal Jump
PT	Plyometric Training
IV	Inverse Variance
SD	Standard Deviation
SRIT	Shuttle-Run Interval Training
S + CDG	Full Squat Combined with COD
RSA	Repeated Sprint Ability
RT	Resistance Training
TRT	Traditional Resistance Training
SG	Strength Group
SMD	Standardized Mean Difference
SRT	Slow-Speed Resistance Training
VG	Velocity Group
